# Characterization of Meningitis and Meningoencephalitis in the Israeli Defense Forces From 2004 to 2015: A Population-Based Study

**DOI:** 10.3389/fneur.2022.887677

**Published:** 2022-06-30

**Authors:** Shany Guly Gofrit, Yoav Yechezkel Pikkel, Hagai Levine, Shifra Fraifeld, Shlomzion Kahana Merhavi, Limor Friedensohn, Ruth Eliahou, Tamir Ben-Hur, Asaf Honig

**Affiliations:** ^1^Medical Corps of the Israel Defense Forces, Haifa, Israel; ^2^Department of Neurology, Hadassah Medical Center, Hebrew University, Jerusalem, Israel; ^3^Braun School of Public Health and Community Medicine, Hebrew University-Hadassah Medical School, Jerusalem, Israel; ^4^Department of Radiology, Hadassah Medical Center, Hebrew University, Jerusalem, Israel; ^5^Division of Endocrinology, Diabetes and Metabolism, Sheba Medical Center, Ramat Gan, Israel

**Keywords:** meningitis, meningoencephalitis, meningitis epidemiology, military medicine, young adults

## Abstract

**Background:**

Meningitis and meningoencephalitis (MME) are potential medical emergencies. Mandatory reporting of all MME cases in the Israel Defense Force (IDF) allows accurate characterization of MME incidence and course. In the present study, we described the epidemiology of MME in soldiers.

**Methods:**

Medical charts of 860,000 combat and non-combat soldiers serving during the years 2004–2015, accounting for 2,256,060 patient years, were retrospectively evaluated. The diagnosis of MME was based on signs of meningeal irritation and a count of > 5 white blood cells (WBC) in the cerebrospinal fluid (CSF). Data on the diagnosis of bacterial or aseptic MME, significant sequelae, and associated mortality were collected.

**Results:**

Approximately 273 cases of MME were diagnosed. Overall, MME incidence was 12.1/100,000 patient-years. Bacterial and viral pathogens were identified in 31/273 (11.4%) and 52/273 (19%) cases, respectively. Combat soldiers had higher incidence of bacterial meningitis [14/40 (35%) vs. 31/212 (14.6%); *p* = 0.002] and meningoencephalitis [13/40 (32.5%) vs. 33/212 (15.6%); *p* = 0.023] compared to non-combat soldiers. Their clinical presentation was more severe, including confusion [10/40 (25%) vs. 22/212 (10.4%); *p* = 0.018], focal neurological deficits [12/40 (30%) vs. 11/212 (5.2%); *p* < 0.0001], and status epilepticus [3/40 (7.5%) vs. 0/212 (0.0%); *p* < 0.01]. Mortality among combat soldiers was higher [5/40 (15%) vs. 1/212 (0.5%); *p* < 0.001]. *N. meningitidis* was the most frequently isolated bacteria, despite universal preventative vaccination.

**Conclusion:**

The incidence of bacterial MME in the IDF is higher than in the civilian population. Combat soldiers present with higher incidence of meningoencephalitis and bacterial meningitis.

## Introduction

Acute bacterial meningitis is a severe disease, with an incidence of about 0.94 cases per 100,000 annually in the Netherlands (1) and a case fatality rate of 14.3% in all age groups (2). Aseptic meningitis and meningoencephalitis (MME) present with a negative bacteria culture, and are often of viral etiology (3). In one report from the United States, incidence of aseptic meningitis was estimated to be as high as 10 per 100,000 per year (4). However, in the United Kingdom, meningitis caused by an identifiable viral agent was estimated to have an annual incidence of 2.73/100,000, suggesting that other etiologies may play a significant role (5).

The Israel Defense Forces (IDF) recruits about half of healthy 18–19-year-old males and females annually for obligatory service of 2–3 years. The IDF mandates reporting of all aseptic and bacterial cases of MME, and reports an average annual incidence for aseptic meningitis of 10.46 per 100,000 (6).

Previous studies have reported that aseptic meningitis is more common in military personnel (7) and soldiers in particular (8). Our experience concurs that there is higher incidence of both meningitis and meningoencephalitis in combat soldiers in data collected in a single medical center (9).

The aims of this study are to provide accurate data on MME in soldiers, and to compare these features in combat vs. non-combat soldiers in the entire military population. We hypothesized that soldiers would have higher incidence of MME compared to civilians, and that combat soldiers will have a more severe clinical course.

## Methods

### Subjects

Medical corps personnel are mandated to report all cases of MME in the IDF to both the IDF public health officer and the chief military neurologist. All cases of suspected infectious meningitis are evaluated by the military public health (MPH) officer and by the Head of the Military Epidemiology Department to exclude non-infectious cases. Every case of infectious MME that is diagnosed during military service is identified and thoroughly investigated. The public health officer confirms the diagnosis of infectious MME, and prospectively records clinical and laboratory data. Details of contagious medical conditions in the soldier's unit are recorded, and appropriate interventions are ordered (Supplementary Figure 1).

All cases of infectious MME for which there was a complete medical record were included in the current study. Cases of MME that had occurred prior to recruitment or during reserve duty after discharge from active duty, and MME due to traumatic, iatrogenic, and other non-infectious causes (for example, inflammatory diseases, such as sarcoidosis, neoplastic diseases, MME associated with trauma, and postoperative MME) were excluded.

The prospective infectious MME database correlating with a source population of 860,000 consecutive soldiers who served on active duty in the IDF from 1.1.2004 to 31.6.2015 was retrospectively reviewed. The year 2004 was chosen as a start year due to the computerization of medical records. The full data set encompasses the equivalent of 2,256,060 patient years for epidemiological purposes. Each patient was followed for a minimum period of 60 days.

The great majority of soldiers were young men aged 18–22 years; however, women serving in the IDF and career military personnel up to 45 years old were also included in the study. IDF soldiers were designated as combat or non-combat according to the post in which they served most of the service period. Some of the soldiers rotated between posts and, therefore, were not defined as either.

### Definitions of MME

Meningoencephalitis was defined as meningitis with signs of parenchymal involvement, such as behavioral changes, confusion, focal cortical deficits, and/or seizures (10).

Bacterial meningitis was defined as follows: LP findings of 100–5,000 cells/μL, mainly neutrophils, protein levels of 100–500 mg/dL, and glucose levels below 40 mg/dL (4, 11). Partially treated bacterial meningitis was defined in cases where the patient was diagnosed and treated in a civilian medical department with documentation of prior antibiotic treatment, a CSF profile including either bacterial characteristics as defined above, or a mixed presentation of both lymphocytes and neutrophils, and a decision to continue antibiotic treatment during hospitalization (12).

Aseptic meningitis was defined as > 5 white cells/mm^3^ in the CSF, with lymphocyte predominance, accompanied by acute symptoms of meningitis with negative gram stain and cultures (13, 14).

Aside from the diagnosis of proven or suspected infectious meningoencephalitis given during hospital admission, each patient record in the cohort was further evaluated by the chief military neurologist and the head of the military epidemiology department in an attempt to exclude non-infectious causes in cases without an identified pathogen. The workup to exclude non-infectious etiologies for aseptic meningitis or meningoencephalitis included CSF pathology, B-cell immunoglobulin gene rearrangement to rule out lymphoma, brain magnetic resonance imaging (MRI) to rule out structural causes, and an immunology panel to exclude meningitis as part of a systemic disease. Paraneoplastic and limbic encephalitis panels were used when clinical suspicion was raised and extended to include additional tests in the more recent years of the study.

### Clinical Data

The complete computerized medical record, including details of all treatments provided at hospitals and clinics across Israel during military service, was reviewed by an experienced military neurologist (AH). MRI images were centrally reviewed by an experienced certified neuroradiologist blinded to the clinical information (RE). Data on medical history, presenting symptoms, time from the first symptom to hospital arrival, management, disease course, laboratory findings (CSF analysis, microbiology, blood biochemistry), and late sequelae were recorded (Supplementary Figure 1). Sequelae were divided into five categories:

- Cognitive impairment based on professional assessment and performance on the Montreal Cognitive Assessment Test (MOCAT), as evaluated by a cognitive neurologist.
- Motor impairment based on assessment in a rehabilitation center.
- Epilepsy based on diagnosis in an epilepsy center.
- Hearing impairment, assessed by an objective hearing test in a hospital or military laboratory.
- Chronic headache requiring treatment on at least three occasions, as recorded by a physician.

A comparison between combat and non-combat soldiers was conducted in all categories.

### Predisposing Factors

A search for predisposing factors was performed for every case of bacterial and parasitic meningitis and included 5–9 complement deficiencies and consultation with an infectious disease specialist for evaluation of immune deficiency. Evaluation of predisposing factors was conducted in patients' computerized medical records.

### Microbiological Methods

Microbiological methods included rapid gram stain, methylene blue stain, thioglycolate, tryptic soy broth, chocolate agar, blood agar, and MacConkey agar medium cultures. Tests for herpes simplex virus (HSV) and enterovirus were performed with polymerized chain reaction (PCR) in all CSF samples. *M. pneumonia*-associated MME was diagnosed by a combination of typical clinical findings in the CSF and positive PCR from a throat swab. Tests for cytomegalovirus (CMV), Epstein-Barr virus (EBV), and varicella zoster virus (VZV) were performed with CSF PCR when requested. In cases where there was a clinical suspicion of West Nile virus (WNV) or sand fly virus (SFV), blood and CSF samples were sent to the zoonotic laboratory of the Central National Virology Laboratories. Staining for cryptococcus was performed using “Indian ink” stain and was further evaluated by PCR for cryptococcal antigen. Evaluation for human immunodeficiency virus (HIV) status was routinely conducted. The patients diagnosed with immune compromise due to any reason were evaluated for opportunistic infections. *Toxoplasma Gondii* was tested by blood serology in every patient with suspected immunocompromised state (a low white blood count or a positive HIV serology) or whenever brain imaging was concerning for cerebral neurotoxoplasmosis.

### Ethical Considerations

The study was approved by the IDF Medical Corps Institutional Review Board (1339-2014). Due to the retrospective nature of the study, informed consent was waived. Data were deidentified.

Potential bias, including differences in report and treatment protocols between civilian hospitals, was minimized by standardization of the military report format for the MME inquiry.

### Statistical Analysis

For categorical variables, a summary is provided, giving sample size and relative frequencies. For continuous variables, summary tables are provided, giving the arithmetic mean (M) and standard deviation (SD). Fisher's exact test was applied to test for relationships between clinical status and quantitative demographic characteristics. A *p* < 0.05 was considered statistically significant. The data were analyzed using SPSS version 23 (IBM SPSS Statistics, Armonk, NY, USA) and GraphPad 8 (Prism, San Francisco, CA, USA).

We used the STROBE cohort checklist when writing our report (15).

## Results

### Demographics

The study population was drawn from a total of 860,000 consecutive soldiers who served for the equivalent a total of 2,256,060 patient years (1,566,350 and 689,710 years for males and females, respectively) in the IDF. A total of 273 soldiers, mean age, 22.4 ± 7.9 years (range, 18–42 years), met inclusion criteria. There were 212 non-combat soldiers, 40 combat soldiers, and 21 soldiers who rotated between combat and non-combat posts and were, therefore, not classified into either group. There were 21/273 (7.7%) soldiers in different stages of basic training compared with 252/273 (92.3%) who had completed basic training (*p* = 0.7).

The overall incidence of MME was 12.1/100,000 patient years (273/225, 6060) and was comparable for combat and non-combat soldiers (*p* = 0.83). Incidences of bacterial meningitis and aseptic meningitis were 2.17/100,000 and 9.39/100,000, respectively; 0.54/100,000 cases included partially treated bacterial meningitis, and two cases from parasitic pathogens.

#### Sex Differences

There were 209/273 (76.6%) males and 64/273 (23.4%) females. Overall, MME incidence was 13.3/100,000 patient years for male soldiers (209/1,566,350) and 9.3/100,000 patient years for female soldiers (64/689,710); *p* = 0.0087. Males had more bacterial meningitis compared to females in both non-office and office positions (*p* = 0.001, *p* = 0.012, respectively).

### Presentation

Presenting symptoms are summarized in Table 1. Compared to non-combat soldiers, combat soldiers presented more often with a reduced level of consciousness, diffuse meningeal irritation signs, and focal motor deficits (*p* < 0.001 for all), as well as confusion and status epilepticus accompanied by cerebral cortical edema (*p* < 0.01 for both).

**Table 1:** Clinical presentation upon arrival at the Emergency Department (ED).

**Sign or symptom**	**Total**	**Combat soldiers**	**Non-combat soldiers**	**p**
	**(*n* = 273)**	**(*n* = 40)**	**(*n* = 212)**	
Time: symptom onset to ED presentation (days)	4.5 ± 6.37	4.8 ± 6.77	4.43 ± 6.29	0.351
Headache	220/273 (80.6%)	31/40 (77.5%)	176/212 (83.0%)	0.499
Fever	163/273 (59.7%)	23/40 (57.5%)	131/212 (61.8%)	0.479
Vomiting	75/273 (27.5%)	11/40 (27.5%)	59/212 (27.8%)	0.847
Photophobia	53/273 (19.4%)	7/40 (17.5%)	45/212 (21.2%)	0.674
Confusion	32/273 (11.7%)	10/40 (25%)	22/212 (10.4%)	**0.018**
Decreased level of consciousness	24/273 (8.8%)	10/40 (25%)	12/212 (5.7%)	**<0.001**
Phonophobia	17/273 (6.3%)	3/40 (7.5%)	14/212 (6.6%)	0.74
Vertigo	17/273 (6.3%)	3/40 (7.5%)	12/212 (5.7%)	0.713
Visual field deficit	13/273 (4.8%)	4/40 (10%)	7/212 (3.3%)	0.063
Seizures	14/273 (5.1%)	5/40 (5%)	9/212 (4.2%)	0.84
Motor deficit	9/273 (3.3%)	6/40 (15%)	3/212 (1.4%)	**<0.001**
Diffuse meningeal irritation signs with positive Kernig and Brudzinski	5/273 (1.8%)	5/40 (12.5%)	0/212 (0%)	**<0.001**
Status epilepticus	3/273 (1.1%)	3/40 (7.5%)	0/212 (0%)	**0.004**
Ataxia	3/273 (1.1%)	2/40 (5%)	1/212 (0.5%)	0.067
Hearing impairment	3/273 (1.1%)	1/40 (2.5%)	2/212 (0.9%)	0.409
focal neuro deficit	23/273 (8.4%)	12/40 (30%)	11/212 (5.2%)	**<0.0001**

*Analysis: Fisher's exact test. p < 0.05 are marked in bold*.

### Pathogens and Sequelae

A pathogen was identified in 85/273 (31.1%) soldiers. Pathogen frequency, common neurologic sequelae, and unique late sequelae are described in Table 2.

**Table 2:** Known pathogens and their sequelae.

**Pathogen**	**Diagnostic test**	**Total** **(*n* = 273)**	**Combat soldiers** **(*n* = 40)**	**Non-combat soldiers** **(*n* = 212)**	**p**	**Common neurological late sequelae (*n*)**	**Unique late sequelae (*n*)**	**Predisposing factors**
*N*.*meningitidis*^b^	G and C	8 (2.9%)	2 (5%)	6 (2.8%)	0.62	Cognitive deficit (4), epilepsy (4)	Neuro-intensive care myopathy with prominent fasciculations (1)	Complement deficiencies in two patients
*S. pneumonia*	G and C	5 (1.8%)	2 (5%)	1 (0.5%)	0.016	Seizures, cognitive deficit (1), Hearing loss (1)		S/P splenectomy in one patient
*S*.*aureus*^a^	G and C	3 (1.1%)	3 (7.5%)	0 (0%)	0.004	Death (3)		
*R. conorii*	ELISA	5 (1.8%)	0 (0%)	5 (2.4%)	0.33	Peripheral vertigo (1), resting tremor (1)		
*M. pneumonia*	G and C	3 (1.1%)	1 (2.5%)	2 (0.9%)	0.41			
*A. baumannii*	G and C	2 (0.7%)	0 (0%)	2 (0.9%)	0.54			
*C. burnetii*	ELISA	2 (0.7%)	0 (0%)	2 (0.9%)	0.54	No sequelae recorded		
*S. milleri*	G and C	1 (0.4%)	0 (0%)	1 (0.5%)	0.66	Multiple brain abscess lead to death		
*S. viridans*	G and C	1 (0.4%)	1(2.5%)	0 (0%)	0.02	Papilledema and bilateral abducens palsy		
*B. henselae*	Serology	1 (0.4%)	0 (0%)	1 (0.5%)	0.66	Optic neuropathy and chorioretinopathy		
Enterovirus	PCR	22 (8.1%)	8 (20%)	14 (6.6%)	0.36	Migraine headache (2)	Unilateral flaccid paralysis (1)	
Varicella zoster virus	PCR	14 (5.1%)	1 (2.5%)	13 (6.1%)	0.37	Moderate unilateral SNHL and peripheral vertigo (1) Radicular pain (1)	Cortical venous thrombosis (1), cognitive impairment (1), trigeminal neuralgia (1)	Adalimumab treatment in one patient
West Nile virus	PCR ELISA	3 (1.1%)	1 (2.5%)	2 (0.9%)	0.41	Unilateral flaccid paralysis (1), cerebellar ataxia (1)	Severe cognitive deficit (1)	
Epstein-Barr virus	PCR	4 (1.5%)	1 (2.5%)	3 (1.4%)	0.62	Fatigue (4), depression (2) subacute cerebellitis (2)		
Cytomegalovirus	PCR	4 (1.5%)	1 (2.5%)	3 (1.4%)	0.62	Epilepsy (1) myopathy with prominent fasciculations (1)		
Herpes simplex virus	PCR	2 (0.7%)	0 (0%)	1 (0.5%)	0.66	Fatigue (1), epilepsy (1)	A subacute relapse of encephalitis (1)	
Mumps virus	IgM serology	1 (0.4%)	0 (0%)	1 (0.5%)	0.66	No sequelae recorded		
Sandfly virus	PCR, ELISA	1 (0.4%)	0 (0%)	1 (0.5%)	0.66	Neuropsychiatric sequelae (1)		
H1N1	PCR	1 (0.4%)	0 (0%)	1 (0.5%)	0.66	No sequelae recorded		
Toxoplasmagondii	CSF, PCR, brain biopsy	1 (0.4%)	0 (0%)	1 (0.5%)	0.66	Cognitive deficit		Hashimotothyroditis
Cryptococcus		1 (0.4%)	0 (0%)	0 (0%)	>0.99			HIV

a *In one patient, S. aureus was only suspected as it was not isolated from the CSF but only from blood and skin cultures*.

b *None of the strains included in the mandatory vaccine received on the enlistment day were identified (A, C, Y, W135 strains)*.

*PCR, polymerase chain reaction; G and C, gram stain and cultures; ELISA, enzyme-linked immunosorbent assay; EBV, Epstein-Barr virus; CMV, cytomegallo virus; WNV, West Nile virus; HSV, herpes simplex virus; SFV, sand-fly virus; SNHL, sensory neural hearing loss*.

*Analysis: Fisher's exact test*.

#### Bacterial Pathogens

*Neisseria meningitidis* was the leading bacterial etiology [8/273, (2.9%)]. Of the eight cases, four were type B, one was type C (in an unvaccinated soldier), and three were not recognized. *N. meningitides* type B continues to be a major cause for meningococcal disease up to this day (three cases during the years 2019–2020, and no cases of other bacterial types, as seen in Supplementary Figure 2). Two out of the eight soldiers were in combat units. All eight presented with either confusion or coma that progressed rapidly over several hours. All the meningococcal patients in this cohort resided in crowded housing situations, which include a high number of soldiers residing in the same room and sharing showers. Prompt epidemiological investigation leading to rapid prophylactic antibiotic treatment in each case prevented disease spread. Infection with *Streptococcus pneumonia* [5/273 (1.8%)] was often preceded by ear pain and a history of otitis media infections. Cases of methicillin-susceptible *Staphylococcus aureus* (MSSA) [3/273 (1.1%)] presented with widespread impetigo outbreak associated with skin lacerations. All three of these patients suffered from severe bacteremia and sepsis and died, including one case where death was attributed to sudden and severe cerebellar edema and uncal herniation. LP was performed in all cases of MSSA and was consistent with findings of bacterial meningitis.

#### Viral Pathogens

Enterovirus was the most common viral pathogen [22/273 (8%)] and was diagnosed in the context of diarrheal epidemic. Enteric cytopathic human orphan (ECHO) virus type B27 was identified in four patients with meningitis who had the disease simultaneously and were from the same platoon of a combat basic training unit that suffered from a concurrent diarrheal epidemic. There were 14/273 (5.1%) VZV cases. The majority of the patients with VZV presented without rash, which led to a delay in identification and treatment, and, possibly, the development of sequelae. There were 4/273 (1.5%) EBV cases. Patients with EBV presented with subacute cerebellitis and were characterized by a slow course of recovery.

#### Fungal and Parasitic Pathogens

One soldier with positive serology for HIV was infected with *Cryptococcus neoformans*, and another soldier with Hashimoto thyroiditis was diagnosed with cerebral neurotoxoplasmosis on imaging and had positive serology for toxoplasma.

### Mortality and Complications

Six of 273 (2.2%) patients from the cohort died because of MME complications; 5/6 (83%) were combat soldiers (*p* = 0.0004). Three deaths (50%) were due to MME of *S. aureus* etiology and one due to *Streptococcus milleri* (Table 2). In the two remaining patients who died, the pathogen was not identified; one case was associated with severe status epilepticus and diffuse brain edema.

### Predisposing Factors

Although tests for HIV were performed in all patients, HIV was a rare predisposing factor, and was positive only in one patient diagnosed with subacute cryptococcal meningitis. In this patient, HIV was diagnosed as part of the evaluation upon admission. Other predisposing factors included occult malignancy, complement deficiencies, status post splenectomy, and immunocompromise due to medications (Table 2). Metabolic disorders (vegetarianism with very low B12 levels) and autoimmune diseases (Hashimoto's thyroiditis, inflammatory bowel disease, lupus) were also identified as predisposing factors.

## Discussion

The incidence of bacterial and aseptic MME was 2.17/100,000, which is more than two times that of the general population (1) (Supplementary Table 1). MME incidence was 13.3/100,000 in males and 9.3/100,000 in females. Bacterial MME incidence was higher in males compared to females in both combat and non-combat roles, possibly due to lower hygiene among males (16), a difference which is more pronounced in the combating units. lower rates of hand washing, coupled with higher tendency to infections (17, 18) may contribute to higher rates of MME in non-combat units.

Combat soldiers also presented with a more severe clinical course and higher mortality rates compared to non-combat soldiers (Table 3). According to the Israeli ministry of health, bacterial meningitis incidence in the general population varied between 0.65–1.6/100,00 during the years 2006–2018; thus, bacterial meningitis was three times more common in soldiers and 138 times more common in combat soldiers, compared to the general population in these years.

**Table 3:** Characteristics of combat vs. non-combat soldiers.

**Criteria**	**Total**	**Combat soldiers**	**Non-combat soldiers**	**p**
	**(*n* = 273)**	**(*n* = 40)**	**(*n* = 212)**	
Aseptic meningitis	212 (77.6%)	26 (65.0%)	170 (80.2%)	**0.039**
Bacterial meningitis	49 (17.9%)	14 (35.0%)	31 (14.6%)	**0.002**
Partially treated BM	10 (3.7%)	0 (0.0%)	10 (4.7%)	0.371
Meningoencephalitis	49 (17.9%)	13 (32.5%)	33 (15.6%)	**0.023**
Brain abscess	1 (0.4%)	0 (0%)	1 (0.5%)	>0.999
Deceased	6 (2.2%)	5 (12.5%)	1 (0.5%)	**0.0004**

*Analysis: Fisher's exact test. p < 0.05 are marked in bold*.

A higher incidence of MME in military personnel has been previously reported (6, 7, 19, 20), (Supplementary Table 1), suggesting that the increase is not due solely to mandatory reporting. Crowded conditions and poor hygiene, both more common among combat soldiers, may contribute to higher incidene (21, 22). Immune supression due to physical (23) and mental stress (24–26), acompanied by sleep deprevation (27, 28), are more severe among combat soldiers, making them prone to MME. Indeed, morbidity and mortality were significantly higher among combat soldiers in the present cohort, although time from the symptom onset to emergency department admission was the same.

Meningococcal disease was present in both combat and non-combat soldiers in our cohort. Presentation of soldiers with meningococcal disease was severe, with deterioration over hours, and a dire prognosis. The rate of meningococcal disease dropped following the introduction of the A, C, Y, and W135 vaccine in the IDF (29); however, there are still reported cases of severe type B meningococcal disease. As mentioned above, the one case of meningococci type C documented after the introduction of routine vaccination occurred in unvaccinated soldiers (Supplementary Figure 2). Vaccination against meningococci type B is reported to be highly effective in preventing outbreaks in universities (30), and simulated models show that the vaccinations are cost-effective in cases of college outbreak (31). Vaccination against type B meningococci should be strongly considered in soldiers, particularly in combat units. No outbreaks are reported in our cohort, supporting the effectiveness of preventing measures applied immediately following diagnosis of meningococcal disease.

*S. aureus* infection is relatively common in soldiers and is more common among combat soldiers who tend to get skin lacerations during training (32). Soft tissue and skin infections in combat soldiers are highly contiguous and are a potential source of an outbreak (33, 34). Three combat soldiers in our cohort had *S. aureus* bacteremia, with seeding of the CNS and meningoencephalitis in the context of an impetigo outbreak. The three patients with meningoencephalitis deteriorated rapidly, leading to their deaths. This severe prognosis is well-described in *S. aureus* bacteremia in general. Furthermore, bacteremia may lead to epidural abscesses, complicating diagnosis and treatment (35–37). Our results support the need for early aggressive treatment in skin infections, and evaluation for suspected abscess in cases of refractory *S. aureus* infection.

Our study have several limitations. It is retrospective in nature, and, therefore, duration of follow-up varied due to differences in the periods of remaining service. Additionally, most of the treatment and diagnostic procedures were conducted in civilian hospitals, excluding only immediate emergency treatment; thus, diagnostic and treatment protocols may be somewhat different from patient to patient, complicating our ability to draw conclusions.

The pathogen was not identified in 69% of patients in the present cohort, compared to other MME cohorts reporting unidentified pathogens in only 37–50% of cases (5, 38, 39). We presume that, as the epidemiological officer was mainly concerned with bacterial infections, follow-up with civilian hospitals to pursue pathogen identification was lacking in cases of aseptic meningitis, resulting in a lower identification rate.

Although diagnostic protocols may differ, all neurology departments involved conducted basic diagnostic procedures aimed at achieving microbiological identification and ruling out non-infectious diagnoses, as can be seen in a previous publication describing the experience of a single medical center (9). Since the population studied is relatively uniform, and since there is a mandatory inquiry following each MME case, biases related to retrospective data collection and differential reporting systems were minimized. Diagnostic procedures conducted during hospitalization are relevant to the years of data collection (2004–2015) and were the most updated standard of care in these years. Newer diagnostic procedures that are used currently may have aided in classification of more of the aseptic MME cases had they been available. Identification of the cause of encephalitis, and especially defining encephalitis as mediated by an infectious agent, presents a diagnostic challenge. A report of encephalitis etiology in the UK by Granerod et al. describes an infectious etiology in 42% of cases, whereas, in 37% of cases, no etiology was identified (38). A systemic review by the same group reports lack of identification of etiology in more than 50% of encephalitis cases across different studies (39), and the clinical resemblance of immune-mediated and infectious encephalitis further complicates accurate estimation of infectious encephalitis incidence (40). The cases defined as infectious encephalitis in the present cohort may thus be an overestimation. Diagnosis was further limited during the collection of the data by the lack of tools that are currently in use for evaluation of immunological etiologies. To minimize misdiagnosis, every case in the present cohort was personally reviewed by both a chief military neurologist and by the epidemiological registry to ensure the best possibility of an accurate diagnosis. Changes in definitions that are relevant to the study design may complicate comparison between the epidemiological data acquired in the present study and the literature. Evaluation for predisposing factors was limited and was mainly performed in cases of bacterial meningitis.

In conclusion, our results present an epidemiological study of all MME cases in the IDF and an estimation of bacterial and aseptic meningitis prevalence in young healthy adults living in crowded conditions. Our results show that bacterial meningitis is more common in soldiers, with a more severe clinical course in combat soldiers. Our findings call for vaccination of this population against type B meningococci. Further research on preventive measures to reduce bacterial meningitis cases is needed. Early intervention and close guidance of an expert epidemiologist may help to prevent outbreaks in other similar crowded accommodations, such as colleges.

## Data Availability Statement

The original contributions presented in the study are included in the article/Supplementary Material, further inquiries can be directed to the corresponding author.

## Ethics Statement

The studies involving human participants were reviewed and approved by the IDF Medical Corps Institutional Review Board (1339-2014). Written informed consent for participation was not required for this study in accordance with the national legislation and the institutional requirements.

## Author Contributions

SG: writing—original draft, validation, formal analysis, and visualization. YP and LF: investigation. HL: writing—review and editing and investigation. SF and SK: writing—review and editing. RE: data curation. TB-H: writing—review and editing and funding acquisition. AH: conceptualization, methodology, writing—review and editing, and supervision. All authors contributed to the article and approved the submitted version.

## Funding

This study was supported by the Milgrom Family Fund (Grant No. 8007511) to TB-H.

## Conflict of Interest

The authors declare that the research was conducted in the absence of any commercial or financial relationships that could be construed as a potential conflict of interest.

## Publisher's Note

All claims expressed in this article are solely those of the authors and do not necessarily represent those of their affiliated organizations, or those of the publisher, the editors and the reviewers. Any product that may be evaluated in this article, or claim that may be made by its manufacturer, is not guaranteed or endorsed by the publisher.
