# Supplementary material for: Characterization of Meningitis and Meningoencephalitis in the Israeli Defense Forces From 2004 to 2015: A Population-Based Study
Source: Front Neurol. 2022 Jun 30;13:887677. doi: 10.3389/fneur.2022.887677 (PMC9279563; doi:10.3389/fneur.2022.887677)
Supplement: Supplementary file 2 [file Data_Sheet_1.pdf]

Supplementary Material

1 Supplementary Figures

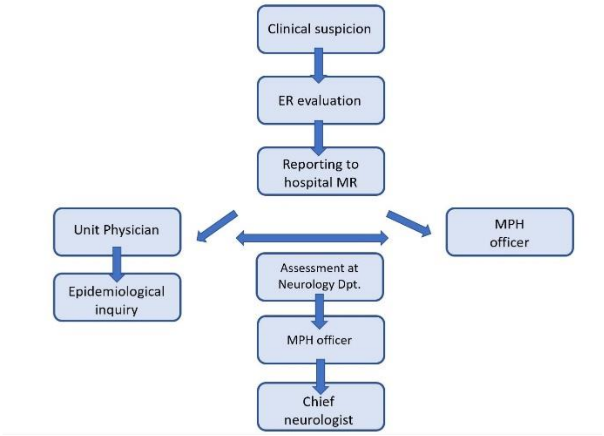

Supplementary figure 1: Reporting chain of events following a suspected MME: in cases of high clinical suspicion for MME, the soldier is evacuated immediately to the nearest ER for further evaluation. Military representatives are present in every medical center in Israel, and they report every MME detail to the MPH officer and the soldier's home unite physician. Debriefing of the soldier's unit includes the order to perform a primary epidemiological investigation, and instructions regarding preventive measures, including collection of samples, and prophylactic treatment. Any developments in the soldier's clinical situation are reported to both the MPH officer and the military chief neurologist. Following evaluations performed by both the military chief neurologist evaluation and the head of epidemiology division, the case is included in the main military epidemiology database.

MR-Military Representative, MPH-Military Public Health

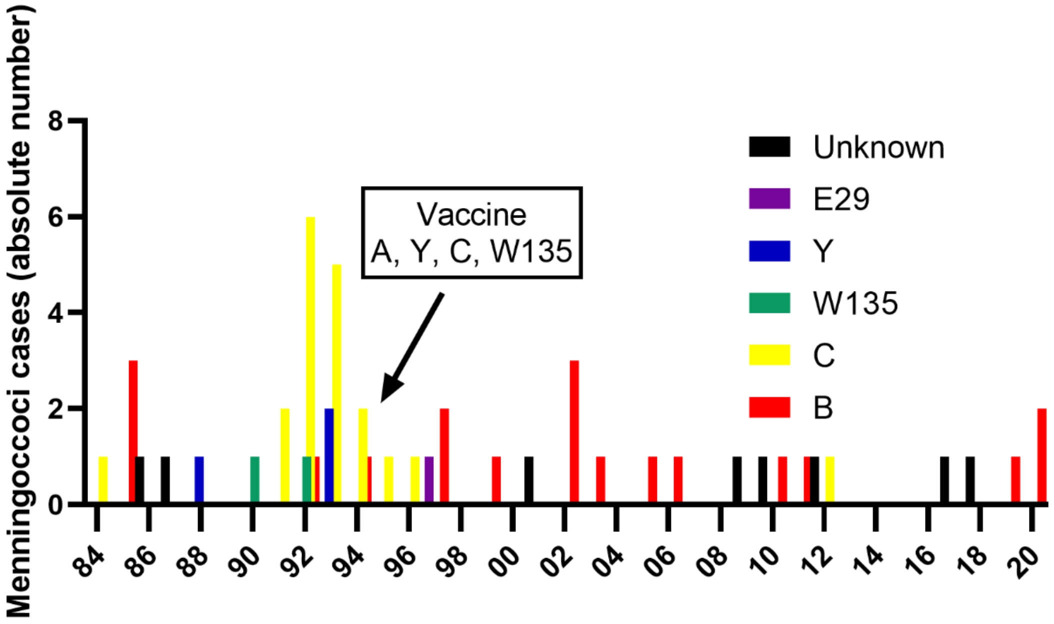

Supplementary Figure 2. Incidence of meningococcal disease divided by type: the meningococcal vaccine, including types A, Y, C, W135 was introduced at 1994. Following vaccination, the main meningococci type observed in soldiers was B.

2 Supplementary table 1. literature review

	Reports in civilians	Reports in military personnel	Present study
**Incidence**	0.94–1.5/100,000 per year for bacterial meningitis (1–3) 10/100,000 per year for aseptic meningitis (4). 2.73/100,000 for meningitis with identified viral pathogen (5)	10.46/100,000 in the years 2003–2012 for aseptic meningitis in the IDF (6).	2.17/100,000 patient years for bacterial meningitis, and 9.39/100,000 patient years for aseptic meningitis.
**Pathogens**	The most common bacterial pathogens are *S. pneumonia*, with incidence of 0.306 per 100,000, followed by *N. meningitidis* with incidence of 0.123 per 100,000 (7).<br><br>Most common viral pathogens are enterovirus and HSV (3,5). enterovirus accounts for about 20%-26% of aseptic meningitis, HSV for 8%-17% and VZV for 8%(5,8).	The most common bacterial pathogens are *N. meningitidis* and *S. pneumoniae* (9,10). Reported incidence for *N. meningitidis* is 0.38–2.2 per 100,000 annually in the US military and the Korean army (11,12).<br><br>The most common viral pathogen is enterovirus (9,10). In a case series from the Naval Medical Center in San Diego, *N. meningitidis* and *S. pneumoniae* each accounted for 1.36% of cases and enterovirus for 19% of cases (9).	The most common bacterial pathogen was *N. meningitidis*, with incidence of 0.35/100,000 patient years (2.9%).<br><br>Most common viral pathogens were enterovirus with incidence of 1/100,000 patient years (12.9% of all aseptic meningitis cases), followed by VZV (8.2%), with HSV accounting for only 1.2% of all aseptic meningitis cases.
**Key sequelae**	Bacterial meningitis lead to hearing loss in 14% of cases, and hemiparesis in 4% (3).<br><br>Overall mortality due to bacterial meningitis is 21% and is higher in *S. pneumoniae* meningitis (13).		Sequelae included hearing loss in two patients, one with *S. pneumoniae* meningitis and one with VZV meningitis.<br><br>Overall mortality due to bacterial meningitis was 4/49(8%).
